# Collectivism/individualism and its relationship to behavioral and physiological immunity

**DOI:** 10.1080/21642850.2014.916218

**Published:** 2014-05-21

**Authors:** Susan G. Brown, Ryan K.M. Ikeuchi, Daniel Reed Lucas

**Affiliations:** ^a^ Department of Psychology, University of Hawaii at Hilo, 200 W Kawili St., Hilo, HI96720-4091, USA

**Keywords:** salivary IgA, collectivism, individualism, hypochondria, perceived vulnerability to disease

## Abstract

The interaction between the behavioral and physiological immune systems provides fertile ground for research. Here, we examine the interactions between fear of disease, collectivism/individualism, disgust, visual perception and salivary IgA. First, we parsed collectivism/individualism into ancestry and psychological processes and examined their relationships to fear of disease. Both ancestral and psychological collectivists scored higher on a test of hypochondria than individualists. Additionally, in two studies we exposed participants to slides of diseased, injured or healthy individuals. Diseased and injured stimuli were rated as equally disgusting, while diseased stimuli were rated as more disgusting than healthy stimuli. We measured salivary IgA in participants before and after they viewed the stimuli. Participants provided information on their ancestral collectivism or individualism. Salivary IgA levels increased after participants viewed images of diseased or injured individuals. Participants with collectivist ancestry tended to react to the diseased and injured images with an increase in IgA, while levels of IgA remained the same or decreased in individualists in one study but we failed to replicate the effect in the second study. An increased salivary IgA response to potentially diseased individuals is adaptive, because salivary IgA plays an important role in protecting individuals from contracting an infection. The response may be related to increased preoccupation with disease states.

## Introduction

One of the most potent agents for natural selection is infectious disease (Anderson & May, 1991). Individuals who survive the pathogens they encounter throughout life are more likely to leave a greater number of offspring than those who die of a disease either prior to or during their reproductive years. In addition, offspring of the former will have a higher probability of immunological protection against the pathogens encountered by their parents. Many factors play a role in the ability of an individual to initially avoid contracting a disease, to recuperate from an infection and to avoid re-infection by a particular pathogen. In fact, one can visualize these factors as four overlapping systems, two behavioral and two physiological. Both the behavioral and physiological immune systems contain a component that minimizes the probability of a person contracting a disease and a component that facilitates the body's assault of microbes or another pathogenic problem.

### Overview of the behavioral immune systems

People engage in a number of behaviors that minimize their contact with potential disease organisms. Specifically, the disgust system is adapted to trigger avoidance of substances like feces, vomit and spit, which have the potential to transmit pathogens (Curtis, de Barra, & Aunger, 2011; Oaten, Stevenson, & Case, 2009). In addition to disgust, some researchers hypothesize that societal collectivism/individualism is associated with pathogen avoidance behavior (Fincher, Thornhill, Murray, & Schaller, 2008; Thornhill, Fincher, Murray, & Schaller, 2010). Collectivist societies consider group membership to be a fixed fact of life, while individualist societies emphasize the self and consider the creation and maintenance of a positive sense of self to be a basic human endeavor (Oyserman, Coon, & Kemmelmeier, 2002). Fincher et al. (2008) found that collectivist societies were historically associated with higher parasite prevalence than individualist societies. They hypothesized that increased parasite prevalence selected for behaviors that facilitated avoidance of individuals who were infected with novel parasitic phenotypes. Additionally, the collectivist insistence on childhood obedience is positively correlated with parasite prevalence (Cashdan & Steele, 2013). Xenophobia, another behavior associated with collectivism, is positively correlated with scores on the Perceived Vulnerability to Disease (PVD) scale. This scale was first developed and tested by Faulkner, Schaller, Park, and Duncan (2004) to measure how susceptible a person felt he was to infection. Navarrete and Fessler (2006) also used the scale and reported that PVD scores were positively correlated with prejudicial attitudes resulting from xenophobia. We explored the relationship between PVD and collectivism/individualism but also wanted to examine whether hypochondriac behavior (HB) which focuses more on the physical aspects of disease than mental fear of disease was associated with collectivist attitudes.

After an individual has contracted a disease, the behavioral immune system acts to maximize behaviors associated with a return to wellness. Illness minimizing behavior initially consists of admittance of illness which, in turn, usually results in behaviors such as resting, taking herbs and/or seeing a medical professional (Woodwell, 1999; Wyke, Hunt, & Ford, 1998). Collectivism also acts to facilitate care-giving behavior in members of the in-group (Fincher et al., 2008).

### Overview of the physiological immune systems

The passive/innate immune system plays a major role in protecting individuals from contracting a disease. The passive immune system consists of physical barriers to pathogens, such as the skin and mucous membranes, as well as chemical barriers, such as complement and lysozymes, along with the lymphocytes (Benjamini & Leskowitz, 1991). Once a disease is contracted the active immune system is triggered. The active/acquired immune system consists, among other things, of B-cells, T-cells and Natural Killer cells (Benjamini & Leskowitz, 1991). Interestingly, the active immune system aids the passive immune system in recognizing previously experienced pathogens through the immunoglobulin system. IgA, which is found in the saliva and mucous membranes, is an especially potent external defense against re-contracting a disease, as well as destroying pathogens with antigens similar to those experienced in a previous illness (Carter & Curran, 2011).

In 2010, Schaller, Miller, Gervais, Yager, and Chen published an article which reported that participants who viewed a slideshow of individuals with visual signs of infection had increased IL-6 levels compared to control individuals who viewed a slideshow of persons carrying and aiming guns. IL-6 is a pro-inflammatory cytokine (Yasukawa et al., 2003) that responds to an internal bacterial trigger. The increased IL-6 response is obviously an adaptive response. Merely viewing the slideshow of diseases sensitized the immune systems of the viewers, increasing their responsiveness to possible infection. If the immune system is capable of responding with an internal increased IL-6 response, we hypothesized that it might also be capable of triggering an immune response that would result in external disease avoidance. An obvious candidate to measure an avoidance response is salivary IgA due to its role in disease avoidance. Salivary IgA attacks microbes prior to their entry into the body. Vaccination data provide a good illustration of the function of salivary and mucosal IgA. For example, vaccines against influenza increased IgA levels in the nasal mucosa (Clements & Murphy, 1986; Samdal et al., 2005; Talaat et al., 2009). Likewise, literature reviews (Carter & Curran, 2011; Tamura & Kurata, 2004) revealed that an increase in IgA levels was a consistent response to vaccination. Some researchers (Hasagawa, Ichinoke, Ainai, Tamura, & Kurata, 2009) have even argued that the induction of mucosal immunity, especially elevated levels of IgA, is a requirement of an effective flu vaccine and effective disease avoidance. In our research, we examined salivary IgA responses to visual stimuli indicating illness and injury.

As is evidenced by the above review, the factors that potentially influence the immune response are complex as are their causal pathways. In the current series of studies, we examine the relationships between visual signs of a diseased or injured state, an early immune response (salivary IgA), the disgust response and collectivism/individualism. As we gathered data on collectivism/individualism in Hawaii it became clear that the concept was multi-faceted. On the one hand, we theorized that an individual could be influenced by her close relatives through both a heritable tendency and through socialization processes resulting in collectivist or individualist behavior (ancestral collectivism/individualism). On the other hand, an individual in a multi-cultural society like Hawaii, that combines elements from both the east and the west, will be confronted with both collectivist and individualist ideals through work, school and other social activities thereby being in a position to combine collectivist and individualist ideas (psychological collectivism/individualism).

In the current series of studies, we tested hypotheses associated with pathogen avoidance. Specifically, in Study 1 we examined ancestral collectivism/individualism, whether an individual's maternal and paternal ancestors were from a collectivist or individualist society, versus psychological collectivism and individualism measured with Triandis’ (1995) collectivism/individualism scale. We examined the relationship between collectivism/individualism and an individual's perceived susceptibility to an illness along with their misperception that they were diseased. The aim of Study 1 was to obtain a better understanding of the relationship between collectivism/individualism and the perceived potential for disease. In Study 2, we examined whether mere exposure to a power point slideshow of diseased versus physically injured individuals resulted in increased levels of salivary IgA. We used sets of stimuli that were rated by independent observers as equally disgusting, and we also examined salivary cortisol levels to test whether stimulus presentations produced a stress response. Additionally, Study 2 examined whether the responses of individuals from predominantly collectivist ancestry differed from individuals from predominantly individualist ancestry. We hypothesized that if collectivist behavior was associated with areas of high parasitism (Fincher et al., 2008), then individuals with more collectivist ancestry should have heightened immune responses to resist parasitism. To clarify, we examined a possible correlational relationship between collectivism and the immune response, not a causal relationship. In Study 3, we tested the same hypotheses but with a non-disgusting control group because the disease and injury slideshows were equally effective in elevating IgA levels in Study 2. The research protocols for all studies were approved by the University of Hawaii Committee on Human Studies.

## Materials, methods and results: Study 1

### Study 1

We explored the relationship between ancestral and psychological collectivism/individualism and health behavior by examining their relationships to perceived susceptibility for disease and hypochondria.

### Participants

The sample consisted of 110 students attending University of Hawaii at Hilo (UHH) and Hawaii Community College (HCC). Participants completed a questionnaire that assessed age, ethnicity of both maternal and paternal grandparents, parents and participant's identified ethnicity. UHH boasts an exceptionally diverse student body, with many individuals of mixed ancestry and diverse ages. Due to this heterogeneity, participants were also assigned an ethnicity percentage. For example, if a participant reported her mother as Japanese, and her father as Caucasian, but identified herself as Japanese, she was coded as Asian, collectivist, and given an ethnicity value of 50%. If the above individual identified herself as Caucasian, however, she would have been coded as Caucasian, individualistic and assigned an ethnicity value of 50%. For certain analyses, we used only individuals of greater than 50% ethnicity. We felt that by concentrating on individuals with greater than 50% ethnicity, we could decrease the variance between individuals thereby increasing our statistical power to reveal differences between collectivists and individualists (Table 1).

**Table 1.** Demographics of participants in the three studies.

	Age	Women	Men	Col.	Ind.	>50% Col.	>50% Ind.
Study 1	23.4 (7.9)	73	37	NA	NA	38	54
Study 2	22.5 (6.8)	28	10	18	20	14	11
Study 3	24.9 (8.5)	60	35	62	33	60	27

Note: Col. indicates individuals classified as collectivists and Ind. indicates individuals classified as individualists. See the text for definitions. The >50% columns contain the numbers of participants with greater than 50% ancestry.

Participants were separated into five ethnic groups: Caucasian (*N* = 37), Asian (*N* = 39), Pacific Islander (*N* = 10), Hispanic (*N* = 4) and African-American (*N* = 2). Africans (Eaton & Louw, 2000) and Hispanics (Hui & Triandis, 1986) are many times classified as collectivists. We, however, decided to classify African-Americans as individualists because Americans, as a whole, are ‘classic’ individualists (Triandis, 1995). Therefore, for the purposes of this study, Caucasians and African-Americans were classified as individualists, while Asians, Pacific Islanders and Hispanics were classified as collectivists. The patterns of statistical significance remained the same even when the Hispanics and African-Americans were eliminated from the analyses; thus, their data were retained for all analyses. For this study, only participants with >50% collectivist or individualist ethnicity were used in the ancestral analyses (Table 1).

### Questionnaires

Cronbach's alpha was used to compute internal consistency of all scales. The psychological collectivism/individualism scale was obtained from Triandis (1995). The 32 questions on Instrument 1 were used, while the scenarios were not. The questionnaire was scored using a 9-point Likert scale with 5 being neutral, 1 being strongly disagree and 9 being strongly agree. There were 17 items used to rate collectivism (for example, ‘My happiness depends very much on the happiness of those around me’) and 15 items used to rate individualism (for example, ‘One should live one's life independently of others’). If a participant failed to answer one item on either sub-scale, the data were not used in analyses involving the scale. This resulted in *N* = 108 for the collectivism sub-scale (*α* = .78) and *N* = 107 for the individualism sub-scale (*α* = .74).

The PVD scale was obtained from Duncan, Schaller, and Park (2009). The 15-item PVD was scored with a 7-point Likert scale with the end points labeled strongly disagree or agree (*α* = .74). This was a different scale than the one used by Faulkner et al. (2004) and Navarrete and Fessler (2006) but was developed by the same experimenters. A factor analysis by Duncan et al. (2009) revealed two factors, one a vulnerability to germs and the other a vulnerability to infection. We also examined the data in terms of the factors (germ *α* = .62; vulnerability *α* = .62). The scale that measured HB was obtained from an online site, Know Your Own Mind (www.tran4mind.u-net.com/hypo.htm). Participants answered 25 questions about their perceptions of their own health in a ‘Yes, Maybe, No’ format. A response of ‘maybe’ was always scored as ‘0’. ‘Yes’ and ‘no’ answers were scored so that the final score represented increased HB (for example, ‘Do you usually feel reasonably well and healthy’ was scored 1 for yes, 0 for maybe and 2 for no but ‘Do you frequently feel faint’ was scored 2 for yes, 0 for maybe and 1 for no). The original internal consistency analysis was low (*α* = .58), so we performed a principal component factor analysis on the 25 questions in the HB scale and reduced the number of questions to the 11 shown in Table 2 (*α* = .62).

**Table 2.** Questions retained and analyzed on the HB questionnaire.

1. Do you worry a lot about catching diseases?
2. Are other people unsympathetic when you are feeling ill?
3. Do you worry a lot about other members of your family getting ill?
4. Do you stay off work if you have any kind of health problem?
5. Do you read medical books and worry that you have all of the symptoms described?
6. Do you find it comforting when you are ill to get all the extra attention and sympathy from your family and friends?
7. Do you feel pity for people who are ill?
8. Are you seldom ill?
9. Do you worry a lot about your health?
10. Do you tend to feel ill around certain people or places?
11. Do you get a deal of pleasure from the health and vitality of your body?

Note: The questions loaded together (loadings ≥ .4) on the first factor in a principal component analysis.

### Results

**Figure 1.**
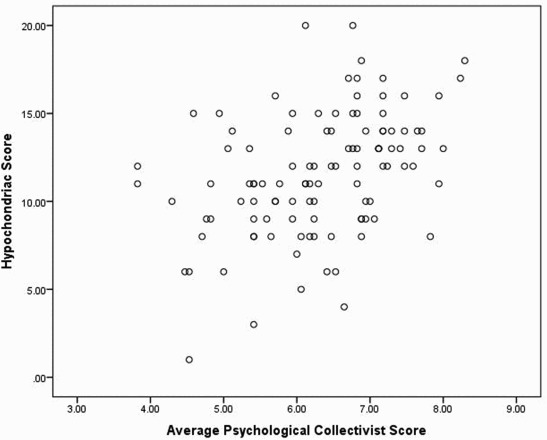
Scatter plot of participants' score on fear of illness as measured by a test of hypochondria and their collectivist scores on Triandis's questionnaire.

**Figure 2.**
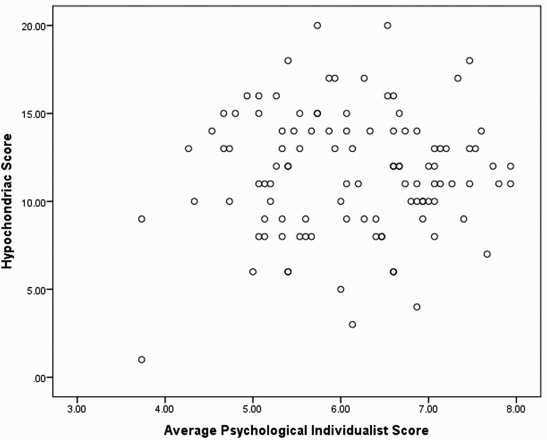
Scatter plot of participants' score on fear of illness as measured by a test of hypochondria and their individualist scores on Triandis's questionnaire.

We used one-way ANOVAs to examine the effects of ancestral collectivism/individualism and gender on the disease vulnerability scales (HB and PVD), and simple correlations to examine the relationships between psychological collectivism and individualism on the scales. Participants with high ancestral collectivism scored significantly higher on the HB scale (MN = 12.56 (SE = 0.47)) than those with high ancestral individualism (MN = 10.47 (SE = 0.53); *F*_(1, 76)_ = 10.47; *p* = .002) but not on the PVD scale (*F*_(1, 76)_ = 1.86; *p* = .18) or either of its factors. Similarly, psychological collectivism was significantly correlated with the HB scale (*r* = .41; *p* < .0001), the PVD scale and to a lesser extent the PVD scales' factors (*r*_PVD_ = .24; *p* = .01; *r*_Infect_ = .20; *p* = .04; *r*_Germ_ = .22; *p* = .02). On the other hand, neither HB (*r* = .04) nor PVD (*r* = −.06) was significantly correlated with psychological individualism. The scatter plots for the correlations of psychological collectivism and individualism with HB are displayed in Figures 1 and 2. We decided to examine the relationship between psychological collectivism/individualism in only individuals with high collectivism/individualism ancestry. The relationships remained the same. In individuals from collectivist backgrounds (*N* = 54), psychological collectivism was related to HB (*r* = .28; *p* = .04) but psychological individualism was not (*r* = −.15; *p* = .27). The same pattern was found for ancestral individualists (*N* = 37); psychological collectivism was correlated with HB (*r* = .38; *p* = .02) but not psychological individualism (*r* = .10; *p* = .56). Age was significantly correlated with PVD (*r* = .20; *p* = .04) and its infect factor (*r* = .22; *p* = .02) but even when age was used as a covariate in the ancestral analysis the relationship remained non-significant (*F*_(1, 78)_ = 2.12; *p* = .14). HB and PVD were significantly correlated (*r* = .32; *p* = .001). Women scored significantly higher on the PVD (MN = 56.6 (SE = 1.44)) than men (50.8 (SE = 2.05); *F*_(1, 108)_ = 6.86; *p* = .01)) and on its infect factor (*F*_(1, 108)_ = 7.58; *p* = .007) but not on the germ factor or psychological collectivism, individualism, HB or age.

## Materials, methods and results: Study 2

### Study 2

Study 2 examined whether exposure to a series of images of diseased versus physically injured individuals would result in increased levels of salivary IgA. Stimuli were rated by independent observers as equally disgusting. Additionally, we examined whether the responses of individuals from predominantly collectivist ancestry differed from individuals from predominantly individualist ancestry. We hypothesized that individuals with more collectivist ancestry should have heightened salivary IgA responses.

### Participants

Data were collected from 38 students attending UHH. After informed consent was provided, participants filled out a questionnaire assessing age, ethnicity of mother and father, and the participant's ethnic identity. Based upon questionnaire data, participants were placed into one of three ethnic groups, Caucasian (*n* = 18), Asian (*n* = 14) or Pacific Islander (*n* = 6). Caucasians were classified as individualists, while Asians and Pacific Islanders were classified as collectivists (Table 1). Procedures for determining ethnicity percentage were the same as Study 1.

### Procedures

Participants were randomly assigned to one of two conditions: the experimental condition, in which participants watched a slideshow of individuals afflicted with visually perceptible diseases (leishmania, trypanosome, malaria, schistosomiasis, filaria, leprosy or spirochetes obtained from Low, 1990), and the control condition, in which participants viewed a slideshow of individuals suffering from injuries unrelated to disease (lacerations, burns or amputation). There were two slides of each of the 7 diseases or injuries, resulting in 14 slides for each condition. Disease and injury images were matched according to afflicted body parts. Each slide was displayed for 5 seconds, and every participant viewed a different random order of the 14 slides. Both the disease and injury slideshows were previously rated as equally disgusting by an independent group of observers (*N* = 55) on a 7-point Likert scale, with 7 being ‘very disgusting’ and 1 being ‘not disgusting at all’ (*M*_Injury_ = 3.5 (SD = 1.56); *M*_Disease_ = 3.4 (SD = 1.19); *F*_(1, 26)_ = 0.07; *p* = .80).

Saliva was collected before and after each slideshow. Participants were asked to rinse their mouths with water prior to collection and then collect saliva through the passive drool method, allowing the saliva to collect on the bottom of their mouths and then easing the saliva into a tube. Participants sat on a futon behind a folding screen to collect saliva and were provided with paper towels to minimize embarrassment. Saliva was stored at −20°C until IgA and cortisol levels could be analyzed through enzyme-linked immunosorbent assay (ELISA). ELISAs were conducted with kits and procedures obtained from *Salimetrics* (see http://www.salimetrics.com for more details). For IgA, the sensitivity was 2.5 µg/mL, the intra-assay coefficients of variation ranged from 4.49 to 6.99 and the inter-assay coefficients of variation ranged from 8.65 to 8.93. For cortisol, the sensitivity was less than 0.003 µg/mL, the intra-assay coefficients of variation ranged from 3.35 to 3.65 and the inter-assay coefficients of variation ranged from 3.75 to 6.41.

### Results

Repeated measures ANOVAs (SPSS 20) were used to examine differences between Groups (Injury versus Disease) and to examine Collectivism/Individualism across the pre- and post-measurements. Simple correlations were conducted between potentially confounding variables. The simple correlations between possible confounding variables found one significant positive correlation between age and experimental IgA levels (*r* = .52; *p* = .001). The other correlations were not significant (*p*s > .31). A repeated measures ANOVA (Ss/Group × Time) with age as a covariate was used to analyze the data on IgA levels. Time of measurement was statistically significant (*F*_(1, 35)_ = 14.2; *p* = .001) but Group (MN_Injury_ = 64.1 (7.4); MN_Disease_ = 62.2 (7.4)) was not (*F*_(1, 35)_ = 0.03; *p* = .86). Participants' salivary IgA rose significantly after they viewed either type of stimuli (MN = 65.8 (6.8)) from baseline levels (MN = 60.6 (5.4)). The interaction between Group and Time was not significant (Injury: MN_Baseline_ = 60.8 (7.8), MN_PostView_ = 67.5 (9.7); Disease: MN_Baseline_ = 60.4 (7.8), MN_PostView_ = 64.1 (9.7); *F*_(1, 35)_ = 0.05; *p* = .82).

**Figure 3.**
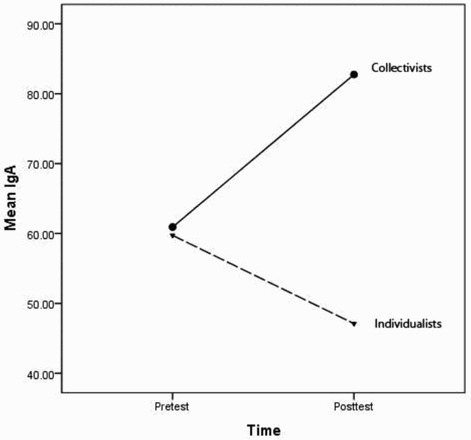
Interaction between ancestral collectivism/individualism and time of saliva collection, prior to observing a slideshow of diseased or injured individuals (pretest) or after (posttest). IgA is measured in μg/mL.

To examine collectivism/individualism and their relationship to change in IgA levels, we performed two analyses. The first analysis used data from all participants' perceived cultural identity and the second analysis used only participants who reported greater than 50% ancestral collectivism/individualism (Table 1). We collapsed the data across the disease and injury conditions because they did not statistically differ from each other. Participants from collectivist backgrounds had higher levels of IgA (MN = 70.3 (6.9)) than participants from individualist backgrounds (MN = 55.3 (7.3)); however, the difference was not significant (*F*_(1, 35)_ = 2.23; *p* = .14). When only participants with greater than 50% ancestry were examined, there was a trend for the Time × Ancestry interaction (*F*_(1, 22)_ = 3.63; *p* = .07). Collectivists displayed increased IgA levels after exposure to the stimuli, while individualists displayed decreased IgA levels (Figure 3). After the interaction was parsed, we found that the collectivists' scores significantly differed from one another in the pre- and posttests (*F*_(1, 18)_ = 47.6; *p* < .0001) but not the individualists' scores (*F*_(1, 16)_ = 1.28; *p* = .28). Cortisol levels did not differ across the time periods, across the groups or across collectivism/individualism.

## Materials, methods and results: Study 3

### Study 3

In Study 2, we controlled the disgust levels in the two sets of stimuli and found no difference between the disease and injury slideshows in eliciting a salivary IgA response. For Study 3, we partially replicated Study 2 but also wanted to use a set of control stimuli that were similar to the disease stimuli but not disgusting. Another difference between the two studies was that Study 2 was conducted in the autumn (September–November) and Study 3 in the winter/spring (February–April).

### Participants

The sample consisted of 95 students attending UHH and HCC. Participants completed a questionnaire identical to the one used in Study 2 that assessed age, ethnicity of parents and participant's perceived ethnicity. Participants were then separated into three primary ethnic groups: Caucasian (*N* = 33), Asian (*N* = 47) or Pacific Islander (*N* = 14). One participant was Iraqi; she was classified as collectivistic, along with the Asians and Pacific Islanders. Caucasians were classified as individualists (Table 1). Procedures for determining ethnicity percentage remained the same as in Study 1.

### Procedures

Participants were randomly assigned to either the experimental or control condition. The experimental (disease) slideshow was not changed from Study 2; however, the control slideshow was replaced with images of visually healthy individuals in settings similar to those in the disease slideshow. Slides were shown in a random order, for five seconds each. In contrast to Study 2, an independent group of observers (*N* = 26) rated the control stimuli (*M*_Control_ = 1.3 (SD = 0.22)) as significantly less disgusting than the disease stimuli (*M*_Disease_ = 3.4 (SD = 1.42); *F*_(1, 26)_ = 35.4; *p* < .0001). Saliva samples were collected before and after participants viewed the slideshow. Samples were treated and analyzed similar to those in Study 2.

### Results

Repeated measures ANOVAs (SPSS 20) were used to examine the differences between Groups (Control versus Disease) and to examine Collectivism/Individualism across the pre- and post-measurements. Simple correlations were conducted between potentially confounding variables. Age was not correlated with IgA levels in Study 3 (*r*_pretest_ = −.01; *r*_posttest_ = .01); therefore, we did not use age as a covariate in the analyses. We conducted the same analyses as in Study 2. Time of measurement was not significant (MN_Baseline_ = 37.3 (2.3); MN_PostView_ = 38.05 (2.4); *F*_(1, 93)_ = 0.15; *p* = .70) nor was the Group effect (MN_Control_ = 36.3; MN_Disease_ = 39.1; *F*_(1, 93)_ = 0.42; *p* = .52) or interaction (Control: MN_Baseline_ = 36.3 (3.3), MN_PostView_ = 36.2 (3.5); Disease: MN_Baseline_ = 38.3 (3.3), MN_PostView_ = 39.9 (3.4); *F*_(1, 93)_ = 0.17; *p* = .68). Additionally none of the main effects or interactions was significant when we examined participants from collectivist versus individualist backgrounds, regardless of whether we used all participants or only participants with >50% ancestral collectivism/individualism backgrounds (*p*s > .20).

## Discussion

People with collectivist ancestry could differ from those with individualist ancestries in their reactions to signs of possible disease in a number of ways. For example, they might display a sensitized disgust response, a larger stress reaction or heightened vigilance. Interestingly, Schaller *et al*. (2010) did not find differences in subjective disgust between their ‘Gun’ and ‘Disease’ groups. In fact, they reported an inverse relationship between changes in stimulated IL-6 levels and disgust. In other words, high IL-6 levels were associated with lower levels of reported disgust although their sample size was small. We did not record the ancestry of the individuals who ranked our visual stimuli for disgust and thus were unable to examine the relationship between collectivism/individualism and disgust. Despite this, it seems unlikely that individuals from collectivist cultures have heightened disgust responses. Stress is also unlikely to differ across the two groups. In our studies, there were no differences in salivary cortisol between the collectivists and individualists.

We found that collectivists scored higher on a questionnaire measuring HB than individualists. This relationship was found whether we examined ancestral or psychological collectivism. Hypochondria is associated with an acute awareness and preoccupation with bodily functions; therefore, this attitude may predispose people to be more aware of, and reactive to, illness symptoms in themselves and others. It could be that hypochondria is simply an extreme form of an adaptive behavioral response. The relationship between collectivism, hypochondria and the immune system is deserving of further study.

Individualists live in areas of the world that host fewer pathogens than the tropics (Fincher et al., 2008). In their places of origin, therefore, they theoretically should be less reactive to pathogens. However, our data indicate that ancestral individualists who display psychological collectivism also have increased hypochondria. It would be interesting to explore this relationship further by testing whether they also have increased immune responses. The results of this study are correlational and therefore we do not know whether hypochondriac individuals are more likely to adopt collectivist attitudes, whether collectivism promotes hypochondria or both are influenced by another variable. Individuals in the current study displayed heightened salivary IgA responses to slideshows of both diseased and injured individuals when compared with control saliva measurements. We used injured individuals as a control slideshow, reasoning that injured persons would elicit helping behavior and, therefore, be more ecologically relevant stimuli than guns. However, we failed to take into account the fact that most of our injured slides portrayed individuals with bloody injuries (10/14 slides) and that blood is a potential medium for infectious disease. Regardless, a heightened salivary IgA response is adaptive to visual elements of both diseased and injured individuals in that it is a potent first line of defense against possible contraction of an infection (Carter & Curran, 2011). In fact, a heightened immune response to blood would protect a caretaker from contracting a blood-borne disease as he or she delivers first aid to the injured person. We failed to replicate these finding in Study 3. At this point, we are unsure whether the failure to replicate occurred because we tested participants from February to April, the height of the Hawaii flu season (Hawaii State Department of Health Disease Outbreak Control Division). The values of salivary IgA were higher in the autumn than in the spring indicating that the energy resources of the participants might have been diverted to alternative immune mechanisms during a time of high disease risk.

In Study 2, people with collectivist ancestry displayed increased salivary IgA to a slideshow of diseased persons while people with individualist ancestry actually showed the opposite response, although the decrease was not significant. Although the simple effects for people from collectivist ancestry differed, there was only a trend for the interaction to be significant so these findings are equivocal. Additionally, we failed to replicate the findings in Study 3. At this point, the conservative conclusion is that there is no relationship between collectivism/individualism and the salivary IgA response. It has been hypothesized that collectivism was initially adaptive for persons living in environments with historically high parasitism rates (Fincher et al., 2008). Individuals who live in environments with high parasitism rates would be expected to have been selected for increased immune responses similar to differences seen in women and men (McClelland & Smith, 2011). Therefore, persons with collectivist ancestry should have heightened immune responses compared to persons with individualist ancestry. This hypothesis needs further research.

The reported studies have several limitations but indicate the direction of future research. First, a more refined way of defining ancestral and psychological collectivism versus individualism would improve the research. One way to accomplish this would be to replicate the research in countries in which collectivists or individualists evolved and compare their responses to second or third generation immigrants of the same ancestry. Additionally, it would be interesting to follow individuals from collectivist and individualist backgrounds after they begin living in multi-cultural settings to examine whether adopting different attitudes toward family or egoism results in behavioral and/or physiological shifts. Second, we did not try to recruit equal numbers of collectivists/individualists because we were using students at UHH and HCC as participants. At this point in time, more women are attending university than men and, in Hawaii, especially at UHH, Asian students outnumber Caucasians. Future research could recruit from a more diverse pool of participants with the aim of achieving more balanced samples. Third, we examined participants at the height of the flu season in Study 3; it would be interesting to examine IgA responses across different time periods and seasons.
